# Understanding cardiovascular healthcare professionals' knowledge and attitudes toward exercise-based cardiac rehabilitation in coronary heart disease

**DOI:** 10.3389/fcvm.2026.1767257

**Published:** 2026-06-10

**Authors:** Hongyan Shi, Hongqiang Zhao

**Affiliations:** 1 Department of Rehabilitation Medicine, TEDA International Cardiovascular Hospital, Cardiovascular Clinical College of Tianjin Medical University, Tianjin, China; 2 Department of Rehabilitation Medicine, TEDA International Cardiovascular Hospital, Tianjin University, Tianjin, China; 3 Department of Medical Insurance, TEDA International Cardiovascular Hospital, Cardiovascular Clinical College of Tianjin Medical University, Tianjin, China; 4 Department of Medical Insurance, TEDA International Cardiovascular Hospital, Tianjin University, Tianjin, China

**Keywords:** cardiac exercise rehabilitationCR, coronary disease, cross-sectional study, health knowledge, attitudes, practice, health personnel, intensive care units

## Abstract

**Introduction:**

This study aims to evaluate the knowledge, attitudes, and practices (KAP) of healthcare professionals in Cardiovascular Medicine, Cardiothoracic Surgery, and Intensive Care Units regarding exercise-based cardiac rehabilitation (CR) for coronary heart disease (CHD).

**Methods:**

This cross-sectional study was conducted between April 2024 and June 2024 at TEDA International Cardiovascular Hospital. Healthcare professionals from Cardiovascular Medicine, Cardiothoracic Surgery, and Intensive Care Units were recruited through convenience sampling via a WeChat-based online questionnaire. A self-developed KAP questionnaire was distributed, and structural equation modeling was used to examine relationships among knowledge, attitudes, and practices.

**Results:**

Analysis of 240 valid questionnaires revealed a response rate of 82%. Among the respondents, 66.7% were female, and 35.4% had 5–10 years of experience. The mean (SD) scores were: knowledge 8.60 (1.20) out of 10, attitude 29.14 (2.15) out of 30, and practice 32.80 (3.77) out of 35. Structural equation modeling indicated that knowledge was directly associated with attitude (*β* = 0.217, *P* = 0.014) and practice (*β* = 0.396, *P* < 0.001), while attitude was also associated with practice (*β* = 0.512, *P* < 0.001).

**Conclusion:**

The findings suggest that healthcare professionals possess adequate knowledge and positive attitudes toward CR for CHD. To improve clinical outcomes, targeted training programs should be established to enhance knowledge and ensure the translation of positive attitudes into practice.

## 1 Introduction

Coronary Heart Disease (CHD) is a leading cause of morbidity and mortality globally. As of 2023, it is estimated that approximately 11.39 million individuals in China are living with CHD. In 2021, the CHD mortality rate among urban residents in China was 135.08 per 100,000 population, while in rural areas, this rate was higher at 148.19 per 100,000 population (1). The impact of CHD is profound, especially in developed nations and regions with aging populations, where lifestyle factors and comorbidities like hypertension and diabetes further elevate risks (2). Exercise-based cardiac rehabilitation (CR) is recognized internationally as a critical component of secondary prevention in CHD management, aimed at improving patient outcomes and reducing mortality and hospital readmissions. CR involves structured physical activity and lifestyle modifications to support cardiovascular recovery, prevent recurrence, and enhance quality of life (3).

The effectiveness of CR is well-supported by global studies and systematic reviews, demonstrating reductions in all-cause mortality and CHD-specific mortality among post-cardiac event patients (4). In recent years, the United States, the United Kingdom, and Australia have expanded CR programs to reach a broader patient population, integrating eHealth and tele-rehabilitation to address accessibility challenges (3–5). However, barriers remain, including limited patient participation rates and variations in program accessibility, especially in developing regions where CR implementation is less comprehensive (6).

The Knowledge, Attitude, and Practice (KAP) model provides a theoretical framework for understanding health behaviors, suggesting that an individual's actions are driven by their knowledge and attitudes. In public health, this model is widely used to assess behavioral practices in conjunction with knowledge and risk perception through KAP surveys, making it highly relevant for analyzing health-related behaviors (7–9).

Healthcare professionals in departments such as Cardiovascular Medicine, Cardiothoracic Surgery, and Intensive Care Units play a crucial role in managing patients with coronary heart disease (CHD) at different stages, from acute care to post-surgical recovery and stabilization. Their responsibilities include initiating CR, educating patients, and developing tailored rehabilitation plans, particularly for critical and high-risk cases where CR is essential. In high-acuity settings such as Cardiothoracic Surgery and ICUs, there is often a perceived tension between maintaining hemodynamic stability and initiating early physical mobilization. While traditional CR focuses on stable outpatients, the “missing link” in the continuum of care lies in the acute transition phase. If healthcare providers in these critical units harbor misconceptions or lack the specific knowledge to prescribe CR safely, the window for optimal recovery may be lost. Thus, assessing the KAP of this specific subset of professionals is not merely academic but a prerequisite for structural clinical reform.

This study aims to assess the KAP of healthcare professionals in these specialized departments regarding CR for CHD, to identify gaps and propose targeted interventions to enhance CR implementation (10, 11).

## 2 Materials and methods

### 2.1 Study design and participants

This cross-sectional study was conducted from April 8 to June 2, 2024, at TEDA International Cardiovascular Hospital. Participants included healthcare professionals from the departments of Cardiovascular Medicine, Cardiothoracic Surgery, and the Intensive Care Unit. The study received ethical approval from the Research Ethics Committee of TEDA International Cardiovascular Hospital ([2024]-0313-1), and informed consent was obtained electronically from participants, allowing only those who consented to proceed with the questionnaire.

Inclusion criteria: (1) healthcare professionals from the departments of Cardiovascular Medicine, Cardiothoracic Surgery, and Intensive Care.

Exclusion criteria: (1) students interning at TEDA International Cardiovascular Hospital; (2) healthcare professionals undergoing further training at TEDA International Cardiovascular Hospital.

The online questionnaire and QR code were created using the Wenjuanxing application (a professional survey platform). The questionnaire was distributed via Wenjuanxing (Changsha Ranxing Information Technology Co., Ltd., Hunan, China), a professional online survey platform. Recruitment was conducted through department directors across 15 departments: 7 Cardiovascular Medicine departments, 6 Cardiothoracic Surgery departments, and 2 Intensive Care Units (CCU and ICU). Department directors distributed the QR code to their respective WeChat work groups, through which all eligible healthcare professionals could access the questionnaire. This sampling approach leveraged the existing hospital communication structure to maximize reach among the target population.

To ensure data quality and completeness, each IP address was limited to a single submission, and all items were mandatory. Members of the research team reviewed questionnaires for completeness, internal consistency, and accuracy. A research assistant was responsible for distributing and collecting the questionnaires, guiding as needed without offering suggestive answers.

### 2.2 Questionnaire design

The questionnaire was designed based on established guidelines and relevant literature (12–15). Experts in CR and statistics reviewed and revised the questionnaire. After three pilot rounds with iterative adjustments, the final questionnaire encompassed four sections (as detailed in the Supplementary File S1): demographic information (education level, gender, job type, institution type, and professional title), knowledge, attitudes, and practices.

The knowledge section encompassed core professional theories, including the fundamental definition of exercise rehabilitation, setting restrictions, individualized patient assessments (e.g., cardiopulmonary exercise testing and heart rate thresholds), and underlying physiological mechanisms (e.g., ischemic preconditioning and the benefits of resistance training). This section comprised 10 items, scoring 1 point for correct and 0 for incorrect/uncertain answers, yielding a total score range of 0 to 10 points. The attitude section reflected the respondents' recognition and willingness to promote exercise rehabilitation, specifically regarding its role in improving patients' quality of life, its integration into routine treatments, the importance of personalized customization, and its positive impact on long-term prognosis. It included 6 items, assessed using a five-point Likert scale ranging from “Very Positive” (5 points) to “Very Negative” (1 point), resulting in a total score range of 6–30 points. The practice section focused on actual clinical behaviors, encompassing active inquiry into patient history, the provision of patient education and guidance, the development of tailored exercise prescriptions, interdisciplinary collaboration, and continuous patient follow-up alongside professional knowledge updating. This section featured 7 items, also scored on a five-point Likert scale from “Always” (5 points) to “Never” (1 point), with a total score range of 7–35 points. Adequate knowledge, positive attitudes, and proactive practices were defined as achieving scores above 70% of the maximum possible score in each respective section (16).

### 2.3 Sample size calculation

Based on Kline's guidelines, the minimum sample size should be at least 10 times the number of predictors. Given that this KAP questionnaire includes 23 independent variables, the required minimum sample size is 230 (17, 18).

### 2.4 Statistical methods

Data analysis was performed using R 4.3.2. Normality tests were conducted for score distributions across each dimension. For normally distributed data, results are presented as means and standard deviations (SD), while for non-normally distributed data, medians and interquartile ranges (25th and 75th percentiles) were used. Categorical data for different demographic characteristics are expressed as N (%). Differences in dimension scores between study groups were analyzed as follows: for normally distributed continuous variables, the *t*-test was applied for two-group comparisons, while the Wilcoxon-Mann–Whitney test was used for non-normal distributions. For comparisons among three or more groups, ANOVA was used for normally distributed data with homogeneous variances, while the Kruskal–Wallis test was employed for non-normally distributed data. Correlations between dimension scores were assessed using Pearson's correlation for normally distributed data and Spearman's correlation for non-normal data. Univariate and multivariate regression analyses were conducted with dimension scores as dependent variables to explore associations with demographic characteristics. Variables with *P* < 0.2 in the univariate analysis were included in the multivariate regression, and results were reported to three decimal places, considering *P* < 0.05 as statistically significant. Based on the KAP theoretical framework, structural equation modeling (SEM) was employed to examine whether attitudes mediate the relationship between knowledge and practice, and to calculate and compare indirect and direct effects. Fit indices for the SEM model were evaluated against threshold criteria: Root Mean Square Error of Approximation (RMSEA) <0.08 as good and <0.10 as moderate (17, 19, 20), Standardized Root Mean Square Residual (SRMR) <0.08, Tucker–Lewis Index (TLI) > 0.8, and Comparative Fit Index (CFI) >0.8. Furthermore, the measurement model component of the SEM mathematically serves as a Confirmatory Factor Analysis (CFA), which validates the construct validity of our self-developed questionnaire.

## 3 Results

### 3.1 Demographic characteristics of participants

A total of 291 questionnaires were collected, with 240 valid questionnaires retained for analysis after data cleaning, leading to an effective rate of 82%. The formal overall Cronbach's *α* coefficient was 0.9026, while the Kaiser-Meyer-Olkin (KMO) value was 0.8721.

Among the 240 respondents, 160 (66.7%) were female, 127 (52.9%) aged 31–40 years, 179 (74.6%) married, 172 (71.7%) held a Bachelor's degree, 125 (52.1%) had an intermediate professional title, 85 (35.4%) had 5–10 years of work experience, and 159 (66.2%) were nurses.

### 3.2 Knowledge, attitudes, and practice scores regarding CR

The mean (SD) scores for knowledge, attitude, and practice were 8.60 (1.20) (possible range: 0–10), 29.14 (2.15) (possible range: 6–30), and 32.80 (3.77) (possible range: 7–35), respectively. Demographic analysis revealed that married participants had significantly lower knowledge scores (*P* = 0.024), while other demographic characteristics did not significantly affect KAP scores (Table 1). The distribution of responses for knowledge items indicated that the highest proportions of participants selecting the “Not Sure” option were for: “The best exercise prescription for CHD patients is based on heart rate, referencing the anaerobic threshold or ischemic threshold.” (K6), with 14.6%; “Patients with CHD must undergo CR exercise therapy in the hospital.” (K2), with 13.3%; and “The optimal daily exercise duration for heart disease patients is:” (K8), with 9.2% (Table 2). In the attitude dimension, over 97% selected “Strongly Agree” or “Agree”, indicating predominantly positive attitudes (Table 3). For the practice dimension, 10% of participants reported only sometimes inquiring about whether CHD patients had received CR during initial consultations (P2), 8.3% sometimes developed personalized CR plans (P4), and 6.7% sometimes provided education and guidance on CR (P3) **(**Table 4).

**Table 1:** Demographic characteristics and KAP scores.

*N* = 240	*N*(%)	Knowledge	*P*	Attitude	*P*	Practice	*P*
		Median [25%,75%] or mean (SD)		Median [25%,75%] or mean (SD)		Median [25%,75%] or mean (SD)	
Total score	240 (100.0)	8.60 (1.20)		29.14 (2.15)		32.80 (3.77)	
Gender			0.771		0.531		0.124
Male	80 (33.3)	8.59 (1.17)		29.08 (2.35)		32.49 (3.78)	
Female	160 (66.7)	8.61 (1.21)		29.18 (2.05)		32.96 (3.76)	
Age			0.324		0.562		0.623
Below 30 years old	71 (29.6)	8.77 (0.94)		28.76 (2.64)		32.59 (3.90)	
31∼40 years old	127 (52.9)	8.47 (1.32)		29.31 (1.95)		32.91 (3.85)	
More than 40 years old	42 (17.5)	8.71 (1.15)		29.29 (1.74)		32.81 (3.32)	
Marital status			0.024		0.873		0.873
Married	179 (74.6)	8.50 (1.25)		29.15 (2.16)		32.80 (3.73)	
Other	61 (25.4)	8.92 (0.95)		29.11 (2.15)		32.79 (3.89)	
Education			0.295		0.159		0.257
Bachelor's degree	172 (71.7)	8.53 (1.27)		29.05 (2.28)		32.90 (3.74)	
Master's degree/PhD	68 (28.3)	8.78 (0.97)		29.38 (1.76)		32.56 (3.84)	
Professional title			0.173		0.256		0.795
Junior	87 (36.2)	8.51 (1.33)		28.67 (2.83)		32.70 (3.76)	
Intermediate	125 (52.1)	8.58 (1.14)		29.41 (1.59)		32.89 (3.83)	
Associate senior/senior	28 (11.7)	9.00 (0.90)		29.43 (1.64)		32.71 (3.59)	
Years of work experience			0.725		0.389		0.578
≤5 years	57 (23.8)	8.70 (1.12)		28.84 (2.61)		32.14 (4.28)	
5–10 years	85 (35.4)	8.48 (1.28)		28.88 (2.47)		32.93 (3.57)	
11–15 years	56 (23.3)	8.66 (1.16)		29.45 (1.58)		32.88 (4.03)	
≥16 years	42 (17.5)	8.64 (1.19)		29.67 (1.05)		33.33 (2.98)	
Doctor or nurse			0.198		0.336		0.338
Doctor	81 (33.8)	8.77 (1.04)		29.33 (1.76)		32.73 (3.64)	
Nurse	159 (66.2)	8.52 (1.26)		29.04 (2.32)		32.84 (3.84)	

**Table 2:** Distribution of knowledge dimension responses.

Knowledge	True	False	Not Sure
1. Cardiac rehabilitation for coronary heart disease refers to improving physical function and cardiovascular condition through exercise training. Exercise therapy is the core component of cardiac rehabilitation.	219 (91.2%)	8 (3.3%)	13 (5.4%)
2. Patients with coronary heart disease must undergo cardiac rehabilitation exercise therapy in the hospital.	88 (36.7%)	120 (50%)	32 (13.3%)
3. Before starting a cardiac rehabilitation program, patients with coronary heart disease should undergo a comprehensive evaluation, including cardiopulmonary function tests and physical condition assessments.	239 (99.6%)	1 (0.4%)	0 (0%)
4. Patients with coronary heart disease need to undergo rehabilitation therapy under the guidance of a doctor.	239 (99.6%)	1 (0.4%)	0 (0%)
5. The maximum exercise intensity threshold for cardiovascular health or fitness benefits must be determined through an exercise stress test.	215 (89.6%)	5 (2.1%)	20 (8.3%)
6. The best exercise prescription for coronary heart disease patients is based on heart rate, referencing the anaerobic threshold or ischemic threshold.	190 (79.2%)	15 (6.2%)	35 (14.6%)
7. Compared to younger coronary heart disease patients, muscle strength training and balance coordination training are more important for elderly coronary heart disease patients.	207 (86.2%)	20 (8.3%)	13 (5.4%)
8. The optimal daily exercise duration for heart disease patients is:	204 (85%)	14 (5.8%)	22 (9.2%)
9. A moderate increase in diastolic blood pressure during resistance exercise is beneficial for improving myocardial perfusion.	212 (88.3%)	8 (3.3%)	20 (8.3%)
10. Aerobic exercise training induces ischemic preconditioning in coronary heart disease patients, enhancing the heart's tolerance to hypoxia, reducing myocardial damage, and lowering the risk of potentially fatal arrhythmias.	220 (91.7%)	7(2.9%)	13(5.4%)

**Table 3:** Distribution of attitude dimension responses.

Attitude	Strongly agree	Agree	Neutral	Disagree	Strongly disagree
I believe that cardiac rehabilitation is an effective method for improving the quality of life of coronary heart disease patients.	197 (82.1%)	36 (15%)	6 (2.5%)	1 (0.4%)	0 (0%)
2. I believe coronary heart disease patients should actively participate in cardiac rehabilitation programs.	212 (88.3%)	25 (10.4%)	3 (1.2%)	0 (0%)	0 (0%)
3. I believe that cardiac rehabilitation should be one of the routine treatments for coronary heart disease.	206 (85.8%)	29 (12.1%)	5 (2.1%)	0 (0%)	0 (0%)
4. I believe that cardiac rehabilitation programs for coronary heart disease patients should be personalized according to individual differences.	219 (91.2%)	19 (7.9%)	2 (0.8%)	0 (0%)	0 (0%)
5. I believe that cardiac rehabilitation has a positive impact on the long-term prognosis of coronary heart disease patients.	215 (89.6%)	21 (8.8%)	4 (1.7%)	0 (0%)	0 (0%)
6. I believe that cardiac rehabilitation should be widely applied.	214 (89.2%)	20 (8.3%)	5 (2.1%)	1(0.4%)	0 (0%)

**Table 4:** Distribution of practice dimension responses.

Practice	Always	Often	Sometimes	Rarely	Never
1. I often recommend cardiac rehabilitation as a treatment option for coronary heart disease patients.	186 (77.5%)	42 (17.5%)	11 (4.6%)	1 (0.4%)	0 (0%)
2. I inquire whether coronary heart disease patients have undergone cardiac rehabilitation during their initial consultation.	176 (73.3%)	39 (16.2%)	24 (10%)	1 (0.4%)	0 (0%)
3. I provide education and guidance to coronary heart disease patients regarding cardiac rehabilitation.	181 (75.4%)	41 (17.1%)	16 (6.7%)	2 (0.8%)	0 (0%)
4. I develop personalized cardiac rehabilitation plans for coronary heart disease patients.	179 (74.6%)	39 (16.2%)	20 (8.3%)	1 (0.4%)	1 (0.4%)
5. I collaborate with rehabilitation therapists or other professionals to provide comprehensive cardiac rehabilitation services for coronary heart disease patients.	189 (78.8%)	44 (18.3%)	6 (2.5%)	0 (0%)	1 (0.4%)
6. I regularly communicate with coronary heart disease patients to monitor their progress and challenges in cardiac rehabilitation.	183 (76.2%)	41 (17.1%)	13 (5.4%)	2 (0.8%)	1 (0.4%)
7. I continuously update and learn about the latest research and guidelines on cardiac rehabilitation for coronary heart disease.	189 (78.8%)	37 (15.4%)	11(4.6%)	2(0.8%)	1(0.4%)

### 3.3 Correlation analysis of KAP dimensions

Correlation analysis showed significant positive correlations between knowledge and attitude (*r* = 0.168, *P* = 0.009), as well as between knowledge and practice (*r* = 0.234, *P* < 0.001). Additionally, a significant correlation was found between attitude and practice (*r* = 0.596, *P* < 0.001) (Table 5).

**Table 5:** Correlation analysis of KAP scores.

Variables	Knowledge dimension	Attitude	Practice
Knowledge dimension	1.000		
Attitude	0.168 (*P* = 0.009)	1.000	
Practice	0.234 (*P* < 0.001)	0.596 (*P* < 0.001)	1.000

### 3.4 Factors associated with KAP scores (regression analysis)

Multivariate logistic regression revealed no factors independently associated with knowledge. However, knowledge was independently associated with a positive attitude (OR = 1.42, 95% CI: [1.11, 1.81], *P* = 0.005). Furthermore, both knowledge (OR = 1.64, 95% CI: [1.23, 2.19], *P* = 0.001) and attitude (OR = 3.52, 95% CI: [1.98, 6.23], *P* < 0.001) were independently associated with proactive practice (Supplementary Table S1).

### 3.5 Structural equation modeling (SEM)

**Figure 1:**
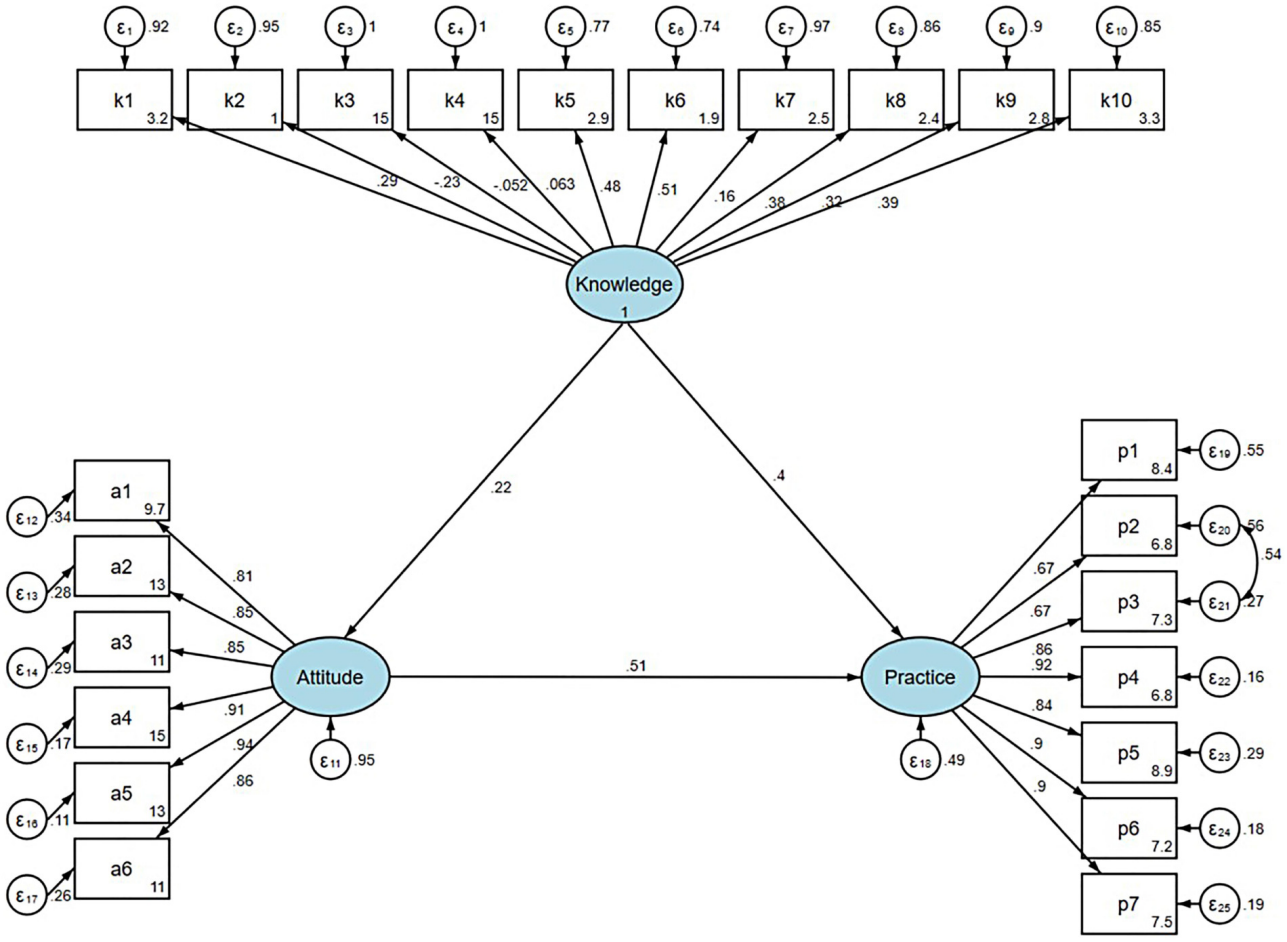
The structural equation model (SEM) rectangle shows observed variables, ellipses indicate potential variables, and circles represent residual terms.

Except for a moderate RMSEA (0.099), the SEM model showed good fit: SRMR = 0.073, TLI = 0.833, and CFI = 0.851 (Table 6). Results showed that knowledge was directly associated with attitude (*β* = 0.217, *P* = 0.014) and practice (*β* = 0.396, *P* < 0.001), while attitude was associated with practice (*β* = 0.512, *P* < 0.001). Additionally, knowledge showed an association with practice (*β* = 0.507, *P* < 0.001) **(**Table 7 and Figure 1).

**Table 6:** The Fit Indices of the Structural Equation Model (SEM).

Indicators	Reference	Results
RMSEA	<0.08 Good	0.099
SRMR	<0.08 Good	0.073
TLI	>0.8 Good	0.833
CFI	>0.8 Good	0.851

**Table 7:** Bootstrap analysis of mediating effect significance test for the final mode.

Model paths		Total effects		Direct Effect		Indirect effect	
		*β*(95%CI)	*P*	β(95%CI)	*P*	β(95%CI)	*P*
Asum							
	Ksum	0.217 (0.044,0.390)	0.014	0.217 (0.044,0.390)	0.014		
Psum							
	Asum	0.512 (0.408,0.616)	<0.001	0.512 (0.408,0.616)	<0.001		
	Ksum	0.111 (0.028,0.194)	0.008	0.396 (0.258,0.534)	<0.001	0.507 (0.361,0.654)	<0.001

## 4 Discussion

Healthcare professionals demonstrated adequate knowledge, positive attitudes, and proactive practices regarding CR for CHD, with significant associations between domains. To further enhance the implementation of CR, targeted educational initiatives should be developed to strengthen healthcare professionals' knowledge, which may be associated with more effective attitudes and practices in the management of CHD patients.

These findings are consistent with similar studies that have reported a generally positive KAP among healthcare workers in cardiac care settings, contributing positively to patient outcomes and adherence to CR programs (21, 22). The positive correlations between knowledge, attitudes, and practices reflect the well-established relationship that higher knowledge levels are associated with more favorable attitudes and better practical application, as supported by prior research (23, 24). SEM results reinforce this link, underscoring associations between knowledge levels with attitudes and practices, consistent with studies suggesting foundational knowledge is critical to behavioral patterns among healthcare professionals.

Marital status was the only demographic factor significantly associated with knowledge scores, with married participants showing lower levels. This result aligns with findings in other studies that suggest married healthcare professionals may have more competing responsibilities outside of work, which could limit opportunities for continuous professional education and skill development (25, 26). The observation that married professionals scored lower in knowledge suggests that the competing demands of work-life balance may hinder long-term professional development. Hospital administrators should consider implementing asynchronous e-learning modules or “micro-learning” sessions that fit into the fragmented schedules of clinicians, rather than traditional, time-intensive seminars. One possible explanation for the uniformity observed in this study could be the structured training programs provided at the participating hospital, standardizing CR knowledge across professional groups. This is further supported by studies showing that standardized training can equalize levels among healthcare professionals (27, 28).

Specific knowledge gaps were identified regarding exercise prescription details, including anaerobic threshold-based prescriptions, optimal daily exercise duration, and diastolic blood pressure adjustments during exercise. These mirror gaps reported in other studies where healthcare professionals lacked detailed knowledge about exercise guidelines and intensity management (29, 30). To address these gaps, it is recommended to implement targeted educational modules that emphasize clinical guidelines for exercise prescription, tailored to the specific needs of CHD patients. These modules should incorporate interactive components such as case-based discussions and hands-on workshops to improve understanding and application. Periodic assessments, including quizzes and practical tests, can reinforce knowledge retention and identify areas requiring further training. It would be beneficial to develop easily accessible resources, such as digital handbooks or mobile apps, summarizing key guidelines for quick reference during clinical practice (28, 31, 32).

Despite positive attitudes toward CR, some hesitation existed regarding its routine integration into standard care. This aligns with findings from other studies where healthcare professionals recognize the benefits but face practical challenges in routine implementation, such as time constraints, lack of resources, or insufficient institutional support (33). To enhance attitudes, addressing these barriers is crucial through organizational changes, such as incorporating CR into institutional protocols and ensuring dedicated time and resources. Establishing a multidisciplinary team including physiotherapists, dietitians, and psychologists can facilitate comprehensive care, making CR more integral to routine CHD management. Moreover, educational initiatives should emphasize not only benefits but also the role in reducing long-term complications and improving quality of life, which may strengthen commitment among healthcare professionals (34, 35).

While proactive behavior was observed overall in the practice dimension, personalized CR planning and patient education showed weaker performance. This trend is consistent with findings from a previous study where personalized care plans and patient education are often hindered by multiple factors among healthcare providers (36). To improve practice, it is recommended to implement mentorship programs where experienced practitioners guide less experienced staff in developing personalized care plans. For example, Johnson et al. developed a service evaluation tool that could help understand and monitor personalized care (37). Additionally, creating standardized templates for individualized CR plans, along with structured patient education materials, can help ensure consistent delivery of care. Regular audits and feedback sessions can be used to monitor CR practices, providing healthcare professionals with insights into areas needing improvement. Integrating regular interprofessional meetings can also promote better collaboration and ensure all team members are aligned in their approach to CR (38, 39).

Specific recommendations to enhance CR delivery should be tailored to different healthcare roles. For doctors, advanced training in exercise prescription and cardiovascular assessments can improve clinical decision-making in CR. For nurses, workshops on motivational interviewing and patient education strategies could enhance communication and patient adherence to CR programs. Providing accessible online courses, particularly for those with limited time due to personal commitments, could help bridge knowledge gaps, as demonstrated in similar studies (40, 41). For patients, Wang et al. showed that patients who underwent PCI had poor knowledge, moderate attitudes, and good practices toward exercise rehabilitation (42). Furthermore, institutional support, such as dedicated CR coordinators or digital tools that facilitate seamless documentation and follow-up, can help integrate CR into routine workflows, thus addressing practical barriers to CR implementation (43, 44).

This study has several limitations. First, this is a single-center study conducted using an online WeChat-based sampling method; the generalizability of the findings to other institutions may be limited, and selection bias may be introduced despite quality control measures such as IP address restrictions and response time screening. Second, the cross-sectional design prevents assessment of causal relationships between knowledge, attitudes, and practices. Third, the self-developed questionnaire, while comprehensive, may have potential biases due to self-reporting and the lack of external validation. Despite these limitations, the study provides valuable insights into healthcare professionals' KAP regarding CR for CHD, highlighting critical areas for targeted educational interventions and practice improvements.

In conclusion, healthcare professionals demonstrated adequate knowledge, positive attitudes, and proactive practices regarding CR for CHD. The observed positive correlations among knowledge, attitudes, and practices highlight the interconnectedness of these factors in promoting effective CR. Enhancing educational initiatives focused on CR may be associated with strengthened healthcare professionals' capabilities, which may contribute to improved patient outcomes in CHD management.

## Data Availability

The original contributions presented in the study are included in the article/supplementary material, further inquiries can be directed to the corresponding author/s.
