# Supplementary material for: Understanding cardiovascular healthcare professionals' knowledge and attitudes toward exercise-based cardiac rehabilitation in coronary heart disease
Source: Front Cardiovasc Med. 2026 Jun 10;13:1767257. doi: 10.3389/fcvm.2026.1767257 (PMC13290582; doi:10.3389/fcvm.2026.1767257)
Supplement: Supplementary file 1 [file Table1.docx]

**Table S1. Univariate and multivariate analysis of knowledge, attitude, and practice**

| **Knowledge** | | | Univariate analysis | | |  | | | Multivariate analysis | |  | |
| --- | --- | --- | --- | --- | --- | --- | --- | --- | --- | --- | --- | --- |
|  |  |  | OR(95%CI) | | | P | | | OR(95%CI) | | P | |
| **Gender** | | |  | | |  | | |  | |  | |
| Male | | |  | | |  | | |  | |  | |
| Female | | | 1.06 (0.59,1.87) | | | 0.844 | | |  | |  | |
| **Age** | | |  | | |  | | |  | |  | |
| Below 30 years old | | |  | | |  | | |  | |  | |
| 31~40 years old | | | 0.64 (0.33,1.21) | | | 0.175 | | | 0.83 (0.37,1.85) | | 0.641 | |
| More than 40 years old | | | 1.03 (0.44,2.50) | | | 0.947 | | | 1.34 (0.49,3.68) | | 0.573 | |
| **Marital status** | | |  | | |  | | |  | |  | |
| Married | | |  | | |  | | |  | |  | |
| Other | | | 1.59 (0.83,3.15) | | | 0.171 | | | 1.52 (0.65,3.55) | | 0.336 | |
| **Education** | | |  | | |  | | |  | |  | |
| Bachelor’s degree | | |  | | |  | | |  | |  | |
| Master’s degree/PhD | | | 1.41 (0.77,2.69) | | | 0.278 | | |  | |  | |
| **Professional title** | | |  | | |  | | |  | |  | |
| Junior | | |  | | |  | | |  | |  | |
| Intermediate | | | 1.02 (0.57,1.83) | | | 0.935 | | |  | |  | |
| Associate senior/senior | | | 1.83 (0.70,5.42) | | | 0.238 | | |  | |  | |
| **Years of work experience** | | |  | | |  | | |  | |  | |
| ≤5 years | | |  | | |  | | |  | |  | |
| 5-10 years | | | 0.82 (0.39,1.68) | | | 0.592 | | |  | |  | |
| 11-15 years | | | 0.98 (0.43,2.19) | | | 0.951 | | |  | |  | |
| ≥16 years | | | 0.95 (0.40,2.28) | | | 0.904 | | |  | |  | |
| **Doctor or nurse** | | |  | | |  | | |  | |  | |
| Doctor | | |  | | |  | | |  | |  | |
| Nurse | | | 0.73 (0.40,1.30) | | | 0.285 | | |  | |  | |
|  | | |  | | |  | | |  | |  | |
| **Attitude** | | Univariate analysis | | |  | | | Multivariate analysis | | |  | |
|  |  | OR(95%CI) | | | P | | | OR(95%CI) | | | P | |
| **Knowledge** | | 1.44 (1.13,1.84) | | | 0.001 | | | 1.42 (1.11,1.81) | | | 0.005 | |
| **Gender** | |  | | |  | | |  | | |  | |
| Male | |  | | |  | | |  | | |  | |
| Female | | 0.73 (0.36,1.42) | | | 0.370 | | |  | | |  | |
| **Age** | |  | | |  | | |  | | |  | |
| Below 30 years old | |  | | |  | | |  | | |  | |
| 31~40 years old | | 1.35 (0.66,2.72) | | | 0.402 | | |  | | |  | |
| More than 40 years old | | 1.15 (0.47,2.99) | | | 0.759 | | |  | | |  | |
| **Marital status** | |  | | |  | | |  | | |  | |
| Married | |  | | |  | | |  | | |  | |
| Other | | 1.10 (0.54,2.35) | | | 0.796 | | |  | | |  | |
| **Education** | |  | | |  | | |  | | |  | |
| Bachelor’s degree | |  | | |  | | |  | | |  | |
| Master’s degree/PhD | | 1.76 (0.85,3.93) | | | 0.145 | | | 1.60 (0.74,3.47) | | | 0.230 | |
| **Professional title** | |  | | |  | | |  | | |  | |
| Junior | |  | | |  | | |  | | |  | |
| Intermediate | | 1.42 (0.74,2.75) | | | 0.291 | | |  | | |  | |
| Associate senior/senior | | 2.03 (0.69,7.47) | | | 0.233 | | |  | | |  | |
| **Years of work experience** | |  | | |  | | |  | | |  | |
| ≤5 years | |  | | |  | | |  | | |  | |
| 5-10 years | | 0.90 (0.40,1.97) | | | 0.795 | | |  | | |  | |
| 11-15 years | | 1.54 (0.61,4.08) | | | 0.368 | | |  | | |  | |
| ≥16 years | | 1.48 (0.54,4.30) | | | 0.454 | | |  | | |  | |
| **Doctor or nurse** | |  | | |  | | |  | | |  | |
| Doctor | |  | | |  | | |  | | |  | |
| Nurse | | 0.71 (0.35,1.39) | | | 0.335 | | |  | | |  | |
| **Practice** | | Univariate analysis | | |  | | | Multivariate analysis | | |  | |
|  |  | OR(95%CI) | | | P | | | OR(95%CI) | | | P | |
| **Knowledge** | | 1.63 (1.28,2.08) | | | <0.001 | | | 1.64 (1.23,2.19) | | | 0.001 | |
| **Attitude** | | 3.40 (1.98,5.85) | | | <0.001 | | | 3.52 (1.98,6.23) | | | <0.001 | |
| **Gender** | |  | | |  | | |  | | |  | |
| Male | |  | | |  | | |  | | |  | |
| Female | | 1.65 (0.95,2.87) | | | 0.074 | | | 1.56 (0.71,3.41) | | | 0.266 | |
| **Age** | |  | | |  | | |  | | |  | |
| Below 30 years old | |  | | |  | | |  | | |  | |
| 31~40 years old | | 1.27 (0.69,2.32) | | | 0.433 | | |  | | |  | |
| More than 40 years old | | 0.87 (0.40,1.89) | | | 0.721 | | |  | | |  | |
| **Marital status** | |  | | |  | | |  | | |  | |
| Married | |  | | |  | | |  | | |  | |
| Other | | 1.06 (0.58,1.96) | | | 0.849 | | |  | | |  | |
| **Education** | |  | | |  | | |  | | |  | |
| Bachelor’s degree | |  | | |  | | |  | | |  | |
| Master’s degree/PhD | | 0.66 (0.37,1.18) | | | 0.157 | | | 0.44 (0.09,2.02) | | | 0.288 | |
| **Professional title** | |  | | |  | | |  | | |  | |
| Junior | |  | | |  | | |  | | |  | |
| Intermediate | | 1.12 (0.64,1.98) | | | 0.684 | | |  | | |  | |
| Associate senior/senior | | 0.81 (0.34,1.96) | | | 0.643 | | |  | | |  | |
| **Years of work experience** | |  | | |  | | |  | | |  | |
| ≤5 years | |  | | |  | | |  | | |  | |
| 5-10 years | | 1.43 (0.72,2.85) | | | 0.305 | | |  | | |  | |
| 11-15 years | | 1.52 (0.71,3.29) | | | 0.280 | | |  | | |  | |
| ≥16 years | | 1.41 (0.62,3.23) | | | 0.415 | | |  | | |  | |
| **Doctor or nurse** | |  | | |  | | |  | | |  | |
| Doctor | |  | | |  | | |  | | |  | |
| Nurse | | 1.48 (0.85,2.56) | | | 0.162 | | | 0.89 (0.20,4.02) | | | 0.878 | |
